# CRISPR-Cas systems: A revolution in genome editing and its diverse applications

**DOI:** 10.46439/biomedres.5.050

**Published:** 2024

**Authors:** Joseph Lin, Jun Yang

**Affiliations:** 1 Department of Internal Medicine, Institute for Human Infections & Immunity, University of Texas Medical Branch, Galveston, TX 77555, USA

**Keywords:** CRISPR-Cas, Genome editing, Agricultural biotechnology, Gene Therapy, Xenotransplantation

## Abstract

The clustered regularly interspaced short palindromic repeats (CRISPR) Cas (CRISPR6 associated protein) system is an advanced adaptive immune system found in prokaryotes. First discovered in1987, CRISPR Cas has revolutionized genetic research in the past two decades. CRISPR-Cas9 the most widespread system enables precise gene editing by creating double strand breaks. Its ease of use and cost-effectiveness has lowered the barrier to entry for genetic research. CRISPR holds immense potential in many fields from agriculture to medicine. In agriculture, CRISPR has accelerated crop improvement by enabling precise gene edits for desirable traits. In medicine, CRISPR holds promise in xenotransplant, cancers and infectious diseases (HIV) treatment. This review traces the historical development of CRISPR-Cas systems, explores their unique applications, and discusses future advancements aimed at enhancing CRISPR’s precision and expanding its applications through technologies like prime and base editing.

## Introduction

Procaryotic organisms are a ubiquitous presence throughout the planet, yet equally as prosperous are phages – viruses that infect procaryotes. This has led to an arms race between procaryotes and viruses. Because of this arms race procaryotes have developed powerful and sophisticated defense mechanisms, including restriction-modification, abortive infection, toxin– antitoxin, and CRISPR–Cas system. The CRISPR-Cas System enables bacteria to incorporate segments of viruses’ DNA into their own genome, creating a biological memory that allows them to target and destroy the specific viruses DNA/RNA in future encounters. However, the CRISPR Cas system isn’t only useful in fending off viruses, but also as a tool to greatly lower the barriers to genetic research. In this review paper, we will delve into the journey of CRISPR-Cas systems, tracing their origins, examining their impact and application in various fields, and exploring their future potential.

## History of CRISPR-Cas System

In 1987, Japanese researchers Yoshizumi Ishino and colleagues at Osaka University discovered a set of repetitive DNA sequences while studying the *E. coli* genome. Initially thought to just be a form of transcription noise, these sequences were later found out to be a part of a larger pattern of repetitive DNA in bacteria. Still despite the observations made, the functions of these sequences remained a mystery for several years [1].

It wasn’t until the 2000s when the function significance of CRISPR sequences began to be discovered. In 2000, Mojica *et al*. identified other clustered repeats in other bacteria and archaea. They coined these sequences as Short Regularly Spaced Repeats (SRSR) [2]. Not long after 2002, Mojica *et al*. coined the term CRISPR referring to the repetitive DNA sequences however the function of CRISPR still remained elusive [3]. By 2005 the function of CRISPR started to be unraveled. Independent groups started to discover that the “spacer” which interspersed the repetitive sequences was derived from foreign DNA, such as phage genomes. This led to the hypothesis that CRISPR was an adaptive immune system [4,5]. The same year Bolotin *et al*. reported an unusual CRISPR locus. While the CRISPR array was similar to those previously reported, it lacked some of the known cas genes which were replaced with novel cas genes. Among these novel cas genes, one encoded for a protein thought to have nuclease activity, now known as Cas9. They additionally noted that the spacers all had a common sequence at one end, this is now known as the protospacer adjacent motif (PAM) [6]. The PAM is crucial for many CRISPR Cas systems as it’s what allows for the system to differentiate between foreign and native DNA. The hypothesis of CRISPR functioning as an adaptive immune system was confirmed by Barrangou *et al*. in 2007 by demonstrating that CRISPR arrays could be updated with new spacer sequences after successful viral infections. This allows bacteria to “remember” and target previously encountered viruses [7].

The following year scientists discovered two important facts: spacer sequences are transcribed into small RNAs (crRNAs) that guide the Cas proteins to the target [8]; CRISPR targets DNA [9]. However, it should be noted that in 2009 it was shown that some CRISPR-Cas systems could target RNA [10]. After the discovery that CRISPR cut DNA, several studies were conducted to study the underlying mechanisms. One example was the discovery of the PAM [11]. Another example was the discovery that an additional small RNA existed, trans-activating CRISPR RNA (tracrRNA), which was used to create the RNA guide for Cas9 [11]. In 2012 two crucial studies were conducted. One characterized Cas9’s mechanisms, showing that Cas9 cleaves the non complementary strand with the RuvC domain while HnH domain cleaves the complementary site. Additionally, they showed that it was possible to reprogram the Cas9 protein to target a site by simply changing the sequence of the crRNA [12]. Subsequently another study in 2012 showed that it was possible to use a single-guide RNA (sgRNA) to replicate the dual crRNA:tracrRNA complex [13].

Soon after these discoveries multiple labs showed that CRISPR-Cas9 could be used to create double strand breaks in DNA and allow for genome editing using DNA repair mechanisms [14,15]. CRISPR’s popularity has exploded as it has greatly increased the accessibility of gene research. Compared to other gene editing techniques, CRISPR doesn’t need years of training or huge amounts of costs. Instead, it only needs some basic training and a modest upfront cost to buy a CRISPR kit [16]. This has allowed for the widespread adoption of CRISPR around the world.

Currently there are two classes of CRISPR: Class 1’s defining feature is their multiple Cas protein effector complex; Class 2’s distinguishing feature is that their effector complex consists of a single multidomain protein. Each class consists of three types with various amounts of subclasses. Class 1 consists of type I, III, and IV; Class 2 consists of type II, V, and VI [17]. Some of the most commonly used systems are Cas9 (type II), Cpf1 (type V), and Cas13 (type VI).

## Applications and Impact of CRISPR

### Applications in agriculture

CRISPR has a wide variety of uses in many fields. In agriculture CRISPR has allowed the potential for rapid improvement. By gene editing crops beneficial characteristics can be amplified, while undesirable traits such as allergen compounds in peanuts can be suppressed. Additionally desirable traits can be introduced into plants such as disease resistance, improved abiotic stress tolerance, etc. For example, in rice by targeting the OsProDH gene with knockout and overexpression the mutation resulted in more proline and less Reactive Oxygen Species (ROS). The OsProDH gene encodes for proline dehydrogenase. Proline plays a large role in getting rid of ROS and protecting the plant from abiotic stresses. Therefore, by mutating the OsProDH gene with CRISPR, the rice can gain greater temperature resistance [18]. However, rice is not the only plant that has been edited using CRISPR, plants such as soybean, potatoes, tomatoes, flax, rapeseed, camelina, and cotton have all also been genetically modified [19]. One notable development is in 2022 pennycress, an oil cash crop gene edited using CRISPR was approved by the FDA. The pennycress was edited to produce less erucic acid and fiber which greatly increased yield and economics of the crop [20].

### Application in xenotransplantation

CRISPR technology is also being explored in xenotransplantation to address the critical shortage of human organs for transplantation. The hope is that CRISPR gene editing could solve the lack of supply by using CRISPR to edit the genes of pigs to allow the pig’s tissues to be compatible with humans. However, there are some hurdles that must be overcome before pig organs can be transplanted into humans.

**Figure 2.**
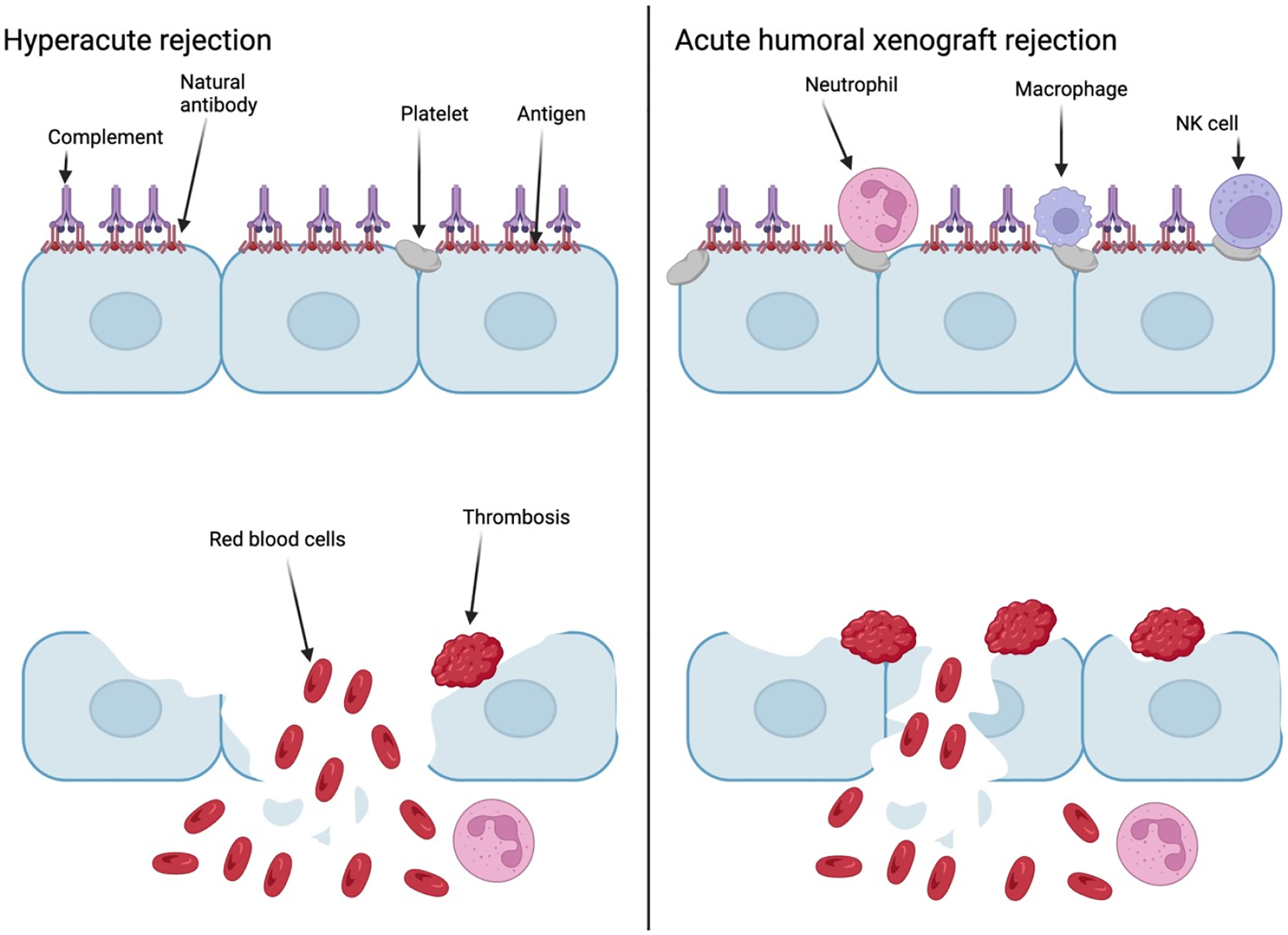
A diagram showing the process and components apart of HAR and AHXR. Created with BioRender.com.

Hyperacute rejection (HAR) happens mere minutes to hours after xenotransplant. HAR is a kind of antibody rection where the antibody of the recipient binds to the epitopes on the porcine endothelial cells (Figure 2), which activates the complement system, leads to the lysis of the endothelial cells and causes the destruction of the grafted vascular system. This subsequently leads to the failure of the transplant. A major antigen that causes HAR is galactose-α1,3- galactose (α-Gal). The epitope is synthesized by alpha-1,3-galactosyltransferase (GGTA1), which is an enzyme encoded by the procine GGTA1 gene. This enzyme is found in most mammals such as pigs, however it is not in humans and other primates. In human blood there is Immunoglobulin G (IgG) which is an antibody that targets α-Gal epitopes. These antibodies are formed by the human body during the neonatal period, bacteria that express the GGTA1 gene exist in the gut. Besides α-Gal there exist two more antigens that also significantly affect occurrence of HAR. The first is cytidine monophospho-N-acetylneuraminic acid hydroxylase (CMAH) that is encoded by the porcine CMAH gene. CMAH is the enzyme responsible for the hydroxylation of N-acetyl neuraminic acid (Neu5Ac) to N-Glycolylneuraminic acid (Neu5Gc). Due to a mutation in the human CMAH gene, Neu5Gc antigen is not expressed. The antibodies against Neu5Gc antigen have been found to be induced after a human eats’ pork. Beta-1,4-N acetylgalactosaminyltransferase 2 (β4GalNT2) is encoded by the porcine β4GalNT2 gene. β4GalNT2 is an enzyme that is involved in the synthesis of the Sd(a) antigen. While humans do have a gene encoding for a homologous enzyme, most people have low levels of anti-Sd(a) IgM antibodies that cause agglutination of red blood cells after blood transfusion from a donor with high expression of the human β4GalNT2 gene and Sd(a) antigen on the surface of cells. Therefore, the inactivation of GGTA1, CMAH, and β4GalNT2 are crucial to overcoming the barrier of HAR [21,22].

Following HAR another hurdle faced by xenotransplant is acute humoral xenograft rejection (AHXR). AHXR happens a few days to weeks after xenotransplant has occurred. AHXR is a type of immune reaction that is caused by antibodies, macrophages, and NK cells. Swine leucocyte antigen (SLA) plays a major role in AHXR, antibodies target SLA that causes adhesion of NK cells and macrophages (Figure 2). This complex then goes to the transplanted organ. The cells will then produce cytokines such as tumor necrosis factor α (TNFα) and interferon γ molecules. This leads to the expression of genes encoding for adhesion and chemotactic factors. The recipient’s platelets then form clots in the small arteries. There are three things that need to happen to prevent AHXR. The first is to modify class I swine major histocompatibility complex (swine leucocytes antigens, SLA) molecules, and disable beta-2- microglobulin (β2M) gene that causes a delayed immune response in the recipient. The second is to reduce the cytotoxicity caused by the recipients NK cells. By eliminating porcine ligands for NK cell activation along with the increased expression of ligands for inhibitory receptors could prevent NK cell caused rejection of xenotransplantation. In addition, directly regulating the NK cells complement inhibition is also an option. By having pigs express the human CD55 (decay accelerating factor) and CD59 genes (membrane reactive lysis inhibitor) protected the cells from the negative effects of the complement system. The final thing is to prevent macrophages from causing toxic effects to the xenografted cells. In order to achieve this, pigs will need to express the human CD47 gene that encodes for integrin associated protein (IAP). IAP is recognized by the human signal-regulatory protein α (SIRPα) found on the macrophage’s cell membrane. When IAP is recognized by SIRPα, it prevents macrophage-mediated autologous phagocytosis [22–24].

Another barrier is Acute cellular rejection (ACR). This immune response usually starts several days after the transplantation. For the xenotransplantation from pig to primates, this type of rejection has rarely been recorded as it’s closely related to the onset of HAR and AHXR. However, due to the advancements that CRISPR has made, multitransgenic animals have been made, which has reduced the problem on HAR and AHXR. Therefore, more and more attention is now being paid towards ACR. CD8+ and CD4+ T cells play the main role in causing ACR. The T cell activation requires a T-cell receptor (TCR) to bind to a major histocompatibility complex (MHC) found on antigen-precenting cells. There are a few ways to prevent T cells from activating. The first way is to express the cytotoxic T cell antigen 4 (CTLA-4) in the pigs. This protein inhibits T cell activity. The second way is to express the human dominant-negative mutant class II transactivator (CIITA-DN) transgene. This is a transactivator of human class II major histocompatibility complex molecules in porcine cells. The third is to switch off the expression of the class I SLA molecules. Both methods resulted in a decrease in porcine antigens on APC cells. If in the future xenotransplantation is to become viable for human use, the CRISPR Cas system will play a major role in this cause. Thanks to the CRISPR Cas system, it has allowed for animals with multiple modifications to be made, which has allowed for the reduction in the reaction of primates’ immune system to porcine cells. Additionally, besides just the immune response, viruses are also a problem that CRISPR has been used to solve. While xenotransplant for humans is still not possible the CRISPR Cas system has brought researchers much closer to this goal [22,25–27].

### Application in HIV treatment

Another interesting application of CRISPR is using it to target the HIV virus. Currently the premier way to treat HIV is combination antiretroviral therapy (cART). cART targets and inhibits the virus in its various stages of the replication cycle. However, cART has multiple drawbacks. Patients must continue cART for the rest of their lives, cART can have side effects, and it’s not widely available around the world. A major barrier for all HIV therapies are HIV reservoirs. These are cells that are infected by the virus and at any time be activated and start virus production once again. Which is why HIV therapy must continue for the patient’s whole life. CRISPR Cas seems to be a promising alternative method to antiviral drugs.

CRISPR Cas has multiple ways that it can treat HIV. The most direct way is to inactive or remove the provirus DNA from the hosts genome. In order to do this, targeting evolutionary constant DNA sequences is the best idea as it will minimize viral escape [28,29]. Researchers using CRISPR Cas9 were able to achieve a 20-fold reduction in HIV virus production and gene expression by targeting the Rev gene in HIV. A less direct way is to use RNA targeting CRISPR such as Cas13a to target viral RNA. While this would suppress the production of more viruses, it fails to address the issue of virus reservoirs. The RNA CRISPRs is not able to target the viral DNA within the hosts genome. This is why for the best results DNA targeting and RNA targeting CRISPRs should be used in tandem to achieve the best results [29–31]. Currently when using Cas9 it can be used to single or multiplex target of viral genes. The single targeting results in a high likelihood of viral escape as DNA repair mechanisms induce mutations, but multiplexing targets is able to avoid this problem [32]. This issue can also be mitigated by switching to a different Cas system. Cas12a doesn’t have the same issues as single targeting Cas9 because Cas12a is able to do multiple cuts due to the cleavage site is quite far from the downstream PAM. These multiple cuts are more likely to introduce detrimental mutations which would prevent viral escape [33]. It’s also important to recognize that CRISPR Cas system could possibly result in off target effects [34]. If the removal of a tumor suppressing gene or the activation of a proto-oncogene could result in uncontrolled cell growth and form tumors. Due to this it is crucial to confirm the stability and safety of a CRISPR Cas HIV treatment [29].

### Application in cancer treatment

**Figure 3.**
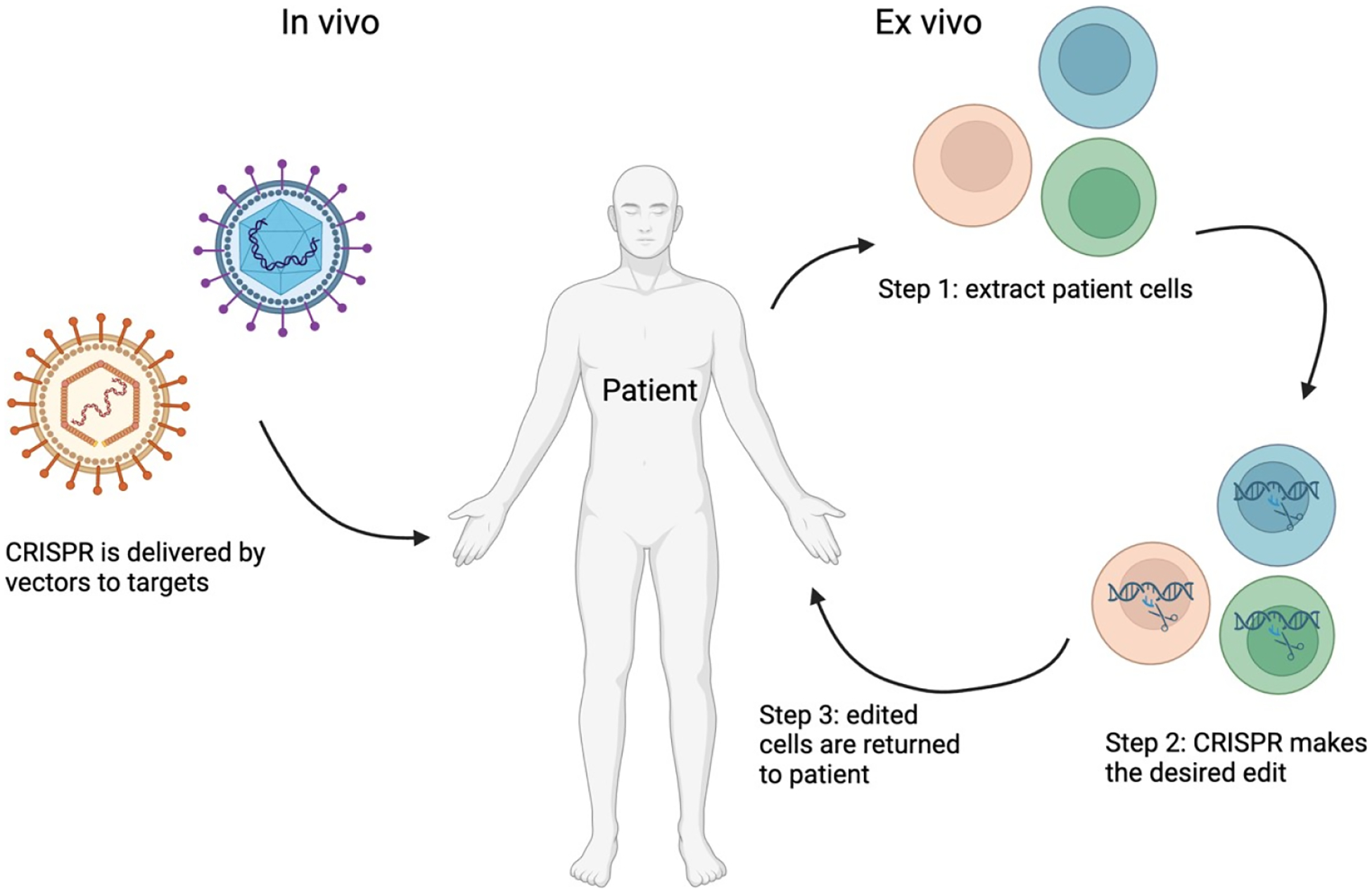
A diagram showing the two methods of CRISPR used in clinical trials. Created with BioRender.com.

Another avenue that is being explored using CRIPSR Cas is cancer treatment. Using CRISPR Cas9 to knockout or knockin genes is proving to be very promising. Besides directly targeting the DNA CRISPR can also be used to edit the cancer epigenome [35,36]. In cancer cells genetic mutations in oncogenes or tumor suppressor genes are present. Using Cas9 attached with a transcription activator or repressor, it’s possible for Cas9 to activate or repress the genes. CRIPSR also serves an important role in immunotherapy. CRISPR can increase the production of chimeric antigen receptor T (CAR-T). Additionally, it was shown that CAR-T cells with their genes that encode for T-cell signaling molecules or inhibitory receptors improved the CAR-T cells ability [37]. Another benefit that CRISPR has brought to immunotherapy is its ability to correct major histocompatibility complex mismatches and aid in the replacement of large MHCs [38]. By knocking out the B2M gene with CRISPR human primary CD4+ T cells were able to lose the expression of the MHC-I surface. This creates a transferable T cell regardless of the antigen genotype. Those with B-cell malignancies have shown to have very positive results due to these universal T cells [39]. CRISPR can also be used to remove inhibitory T-cell surface receptors such as programmed cell death protein 1 (PD-1), which increase the T-cells ability to fight cancer [36]. Currently in clinical trials there are two approaches used using CRISPR (Figure 3).

## Conclusion and Future

CRISPR has revolutionized the field of genetic research. It offers unprecedented precision and versatility when it comes to gene editing, all the while being much easier and cheaper to use than previous methods. CRISPR has shown itself to have immense potential in all sorts of fields ranging from agriculture to medicine. In agriculture. CRISPR is being used to target specific genes to increase crops resistant to diseases, pests, abiotic stresses, etc. In addition to application for agriculture, one of the most significant contributions of CRISPR has been in the realm of medical research and treatment. By enabling precise modifications to the human genome, CRISPR has allowed for the development of innovative therapies for genetic disorders, cancers, and infectious diseases [40]. Furthermore, CRISPR has allowed for the improvement of immunotherapies, such as the improved engineering of CAR-T cells which have shown substantial promise [36].

Despite its numerous advantages, CRISPR is not without issues. Off-target effects, where unintended genetic modifications occur remain a significant concern. These unintended edits can lead to horrible consequences. For example, the activation of oncogenes or the suppression of tumor suppressor genes could potentially lead to cancer. Therefore, ensuring the preciseness and safety of CRISPR based treatments is crucial [29].

Looking into the future, research and advancements should improve CRISPR’s precision and increase its versatility. New techniques such as prime editing and base editing [41,42] will improve the accuracy of CRISPR while also reducing the chances of off-target effects.

Additionally, the research into other CRISPR variants besides Cas9, such as Cas12, Cas13, and Csm [43–45], will expand the versatility of CRISPR.

CRISPR has proven to be a golden goose that keeps giving. While challenges remain, the continued refinement and research of CRISPR are expected to address some of the most pressing challenges of our time. The future holds great promise for even more significant advancements in the years ahead.

## Figures and Tables

**Figure 1.**
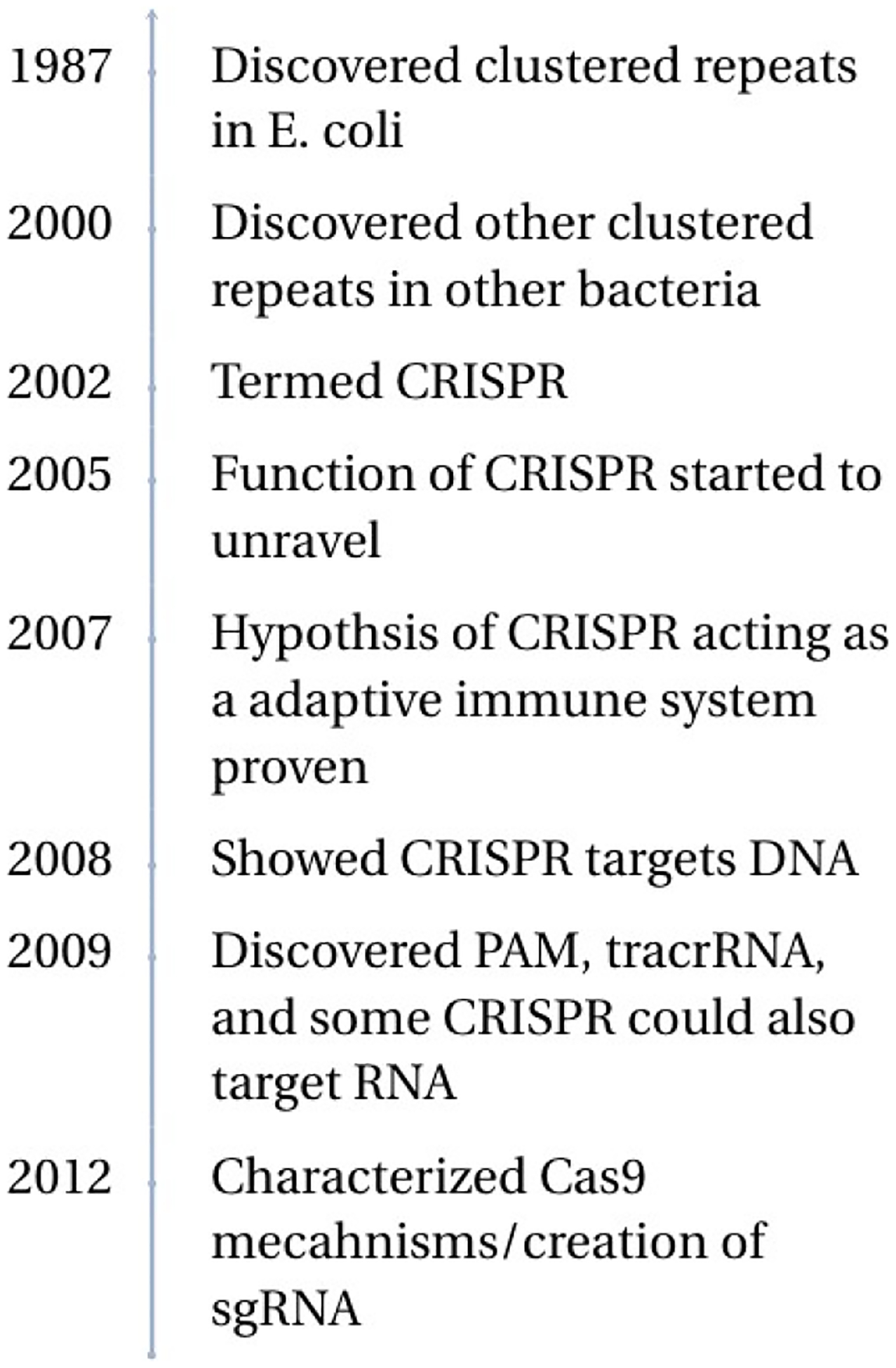
History of CRISPR-Cas System.

## Abbreviations:

CRISPR: Clustered Regularly Interspaced Short Palindromic Repeats
Cas: CRISPR associated protein
PAM: Protospacer Adjacent Motif
crRNA: CRISPR RNA
tracrRNA: Trans Activating CRISPR RNA
sgRNA: Single-guide RNA
Cpf1: CRISPR from Prevotella and Francisella 1 (also known as Cas12a)
ART: Antiretroviral Therapy
HAR: Hyperacute Rejection
AHXR: Acute Humoral Xenograft Rejection
ACR: Acute Cellular Rejection
GGTA1: Alpha-1,3- galactosyltransferase (gene)
CMAH: Cytidine monophospho-N-acetylneuraminic acid hydroxylase (gene)
β4GalNT2: Beta-1,4-N-acetylgalactosaminyltransferase 2 (Gene)
CAR-T: Chimeric Antigen Receptor T-cell
ROS: Reactive Oxygen Species
Rev: Regulator of Expression of Virion Proteins (HIV Gene)
